# Productive and Non-productive Pathways for Synaptotagmin 1 to Support Ca^2+^-Triggered Fast Exocytosis

**DOI:** 10.3389/fnmol.2017.00380

**Published:** 2017-11-15

**Authors:** Jaewook Kim, Yeon-Kyun Shin

**Affiliations:** Roy J. Carver Department of Biochemistry, Biophysics and Molecular Biology, Iowa State University, Ames, IA, United States

**Keywords:** SNARE proteins, synaptotagmin 1, membrane fusion, supported bilayer, single molecule biophysics, exocytosis

## Abstract

Ca^2+^-triggered SNARE-mediated membrane fusion is essential for neuronal communication. The speed of this process is of particular importance because it sets a time limit to cognitive and physical activities. In this work, we expand the proteoliposome-to-supported bilayer (SBL) fusion assay by successfully incorporating synaptotagmin 1 (Syt1), a major Ca^2+^ sensor. We report that Syt1 and Ca^2+^ together can elicit more than a 50-fold increase in the number of membrane fusion events when compared with membrane fusion mediated by SNAREs only. What is remarkable is that ~55% of all vesicle fusion events occurs within 20 ms upon vesicle docking. Furthermore, pre-binding of Syt1 to SNAREs prior to Ca^2+^ inhibits spontaneous fusion, but intriguingly, this leads to a complete loss of the Ca^2+^ responsiveness. Thus, our results suggest that there is a productive and a non-productive pathway for Syt1, depending on whether there is a premature interaction between Syt1 and SNAREs. Our results show that Ca^2+^ binding to Syt1 prior to Syt1's binding to SNAREs may be a prerequisite for the productive pathway. The successful reconstitution of Syt1 activities in the physiological time scale provides new opportunities to test the current mechanistic models for Ca^2+^-triggered exocytosis.

## Introduction

One of the truly remarkable features of the neuron is its ability to release neurotransmitters in <1 ms, in response to the Ca^2+^ influx (Schneggenburger and Neher, 2000; Burgalossi et al., 2012). Neurotransmitter release is achieved by means of synaptic vesicle fusion onto the plasma membrane. The protein components that mediate fast membrane fusion have largely been identified and most of them are well-characterized biochemically and structurally. The highly conserved soluble N-ethylmaleimide-sensitive factor attachment protein receptor (SNARE) proteins are the central fusogen (Südhof and Rothman, 2009). Cognate vesicle (v-) SNARE and target plasma membrane (t-) SNAREs associate to form a four-stranded coiled coil which drives membrane fusion (Poirier et al., 1998; Sutton et al., 1998; Weber et al., 1998). Additionally, synaptotagmin 1 (Syt1) is known to be a major Ca^2+^-sensor (Brose et al., 1992; Fernández-Chacón et al., 2001; Chapman, 2008) while complexins (Cpx) (Giraudo et al., 2006; Tang et al., 2006; Xue et al., 2007) and sec1/Munc18-like (SM) (Südhof and Rothman, 2009; Ma et al., 2013) proteins are both believed to play intimate roles in tightly regulating membrane fusion. However, the mechanisms whereby these protein components orchestrate synchronized vesicle fusion in such a short time scale is still unclear at the molecular level.

An effective and powerful approach to delineate the mechanism may be the *in vitro* reconstitution of the membrane fusion reaction. For example, fusion of v-SNARE-reconstituted proteoliposomes to t-SNARE-containing supported bilayer (SBL), when analyzed with single molecule spectroscopy, revealed that SNAREs alone are capable of mediating membrane fusion in <25 ms (Liu et al., 2005; Domanska et al., 2009; Kiessling et al., 2013). However, attempts to functionally incorporate Ca^2+^-sensor Syt1 using this platform have not been successful. Thus, it was impossible to test the proposed mechanistic models of Ca^2+^-triggered exocytosis with this method.

Meanwhile, an alternative experimental platform which monitors fusion between two proteoliposomes has proven effective in dissecting the functions of individual protein components. This approach has been used to demonstrate that SNARE proteins are the core fusion machinery (Weber et al., 1998; Yoon et al., 2006), that Syt1 is the Ca^2+^-sensor (Lee et al., 2010; Kyoung et al., 2011), that Cpxs are the clamping agent for spontaneous fusion (Schaub et al., 2006) and that Munc18 is part of the core fusion machinery (Shen et al., 2007). It has also been shown that Munc13 is a critical component for quality-controlling t-SNAREs to be ready for productive membrane fusion (Liu et al., 2016). However, when fusion between two single proteoliposomes was analyzed with single molecule technique, it turned out that the speed of membrane fusion was in the time scale of several seconds (Lai et al., 2013), as much as four orders of magnitude slower than the physiological rate. Such slow speed raises concerns of some critical, missing components which are not incorporated in the assay or whether the proteoliposome fusion assay does not faithfully reproduce vesicle fusion *in vivo*. Some suspect that the discrepancy might be due to the tight membrane curvature of proteoliposomes which may not closely mimic the relaxed curvature of the plasma membrane. Others wonder if our long-standing dogma that SNAREs are the core membrane fusion machinery is valid (Wickner and Rizo, 2017).

In this work, we expand the proteoliposome-to-SBL fusion assay by successfully incorporating Syt1. We observe a more than 50-fold increase in the number of membrane fusion events in the presence of Ca^2+^ when compared with those without Syt1. Most importantly, more than ~55% of all vesicle fusion occurs within our instrumental time limit, 20 ms, after docking to the bilayer surface. Further analysis reveals that Syt1 binding to t-SNAREs prior to Ca^2+^ clamps spontaneous fusion. However, this pre-binding leads to a failure to respond to Ca^2+^ in promoting synchronized membrane fusion. Thus, our results show that there may be productive and non-productive pathways for Syt1 in supporting fast membrane fusion. We then suggest possible mechanisms whereby Syt1 might be steered to the productive pathway. Importantly, the improved membrane fusion assay provides new opportunities to test the mechanistic models for Ca^2+^-triggered exocytosis in a time scale ever closer to the physiological one.

## Results

### SNAREs are capable of driving fast membrane fusion

Previously, fast fusion between v-SNARE-reconstituted vesicles (v-vesicles) and t-SNARE-reconstituted supported bilayers (t-SBL) was successfully demonstrated (Liu et al., 2005; Domanska et al., 2009; Karatekin et al., 2010; Kiessling et al., 2013; Stratton et al., 2016). This environment displayed the fusion kinetics that better mimicked what was observed *in vivo*. The results suggested that SNAREs alone, without the help of any auxiliary proteins, are capable of driving sub 25 ms membrane fusion.

**Figure 1:**
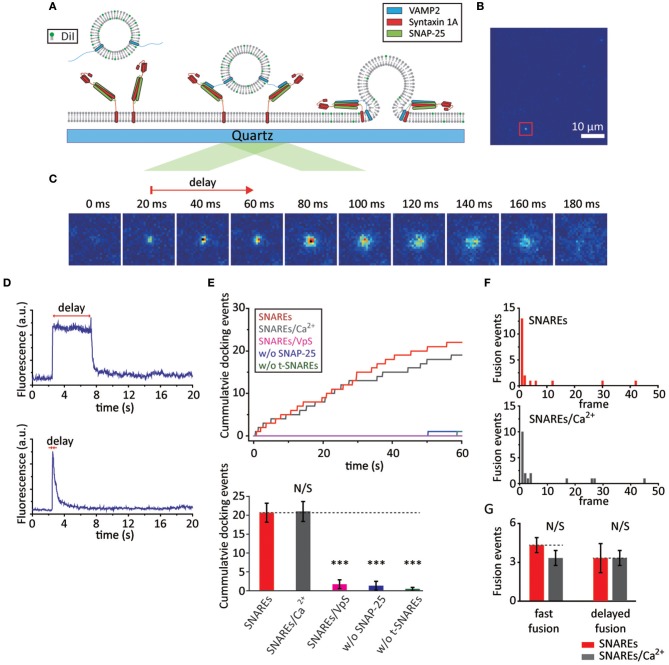
SNAREs are capable of mediating fast membrane fusion. **(A)** Schematics for the single vesicle-to-SBL fusion assay. **(B)** Screen shot (55 × 55 μm) of the video recording. The red box indicates a docked vesicle. **(C)** Time lapse of a single membrane fusion event; each image is 20 ms apart. The red arrow indicates the time delay between docking and the start of fusion. The time delay here is 40 ms. **(D)** Time traces of slow (upper) and fast (below) membrane fusion events are shown. **(E)** Docking events with SNAREs only (red) and SNAREs with 500 μM Ca^2+^ (gray). The controls with t-SBL deactivated by VpS (magenta), with t-SBLs constructed without SNAP-25 (blue), and without both SNAP-25 and syntaxin 1A (green) are also shown. Representative traces of cumulative counts of docking against time (upper) and the number of docked vesicles during the 1 min observation period. We observed a total of 62, 63, 5, 4, and 1 docking events for SNAREs, SNAREs/Ca^2+^, SNAREs/VpS, w/o SNAP-25, and w/o t-SNAREs, respectively. **(F)** A representative histogram of docking-to-fusion delay distribution with only SNAREs in the absence (upper) and presence of Ca^2+^ (below). We observed a total of 23 and 20 number of fusion events in the absence and presence of Ca^2+^ from three independent video recordings, respectively. Each frame is 20 ms apart. **(G)** The number of fusion events occurring within the 1^st^ frame (fast fusion) and the sum of those occurring thereafter (delayed fusion) within a 1 min recording. For **(E)** and **(G)** the data are shown as means ± *SD*. Statistical significance was assessed by Student's *t*-test (^***^p < 0.005; NS, no significant difference; *n* = 3 independent experiments).

To investigate SNARE-mediated single vesicle-to-SBL fusion in our hands, we prepare the t-SBL with PEGylated liposomes following the method previously reported by Karatekin et al. (2010). It has been shown that the PEGylated lipids provide a hydrated cushion of ~4 nm in thickness between the SBL and the imaging surface which allows for critical dynamic movement of transmembrane proteins (Karatekin and Rothman, 2012). Once the SBL is properly formed we place the slide under the total internal reflection fluorescence (TIRF) microscope and start video acquisition as we introduce the v-vesicles (Figure 1A). The v-vesicles contain lipophilic cationic fluorescent dye 1,1′-dioctadecyl-3,3,3′,3′-tetramethylindocarbocyanine perchlorate (DiI) (excitation at 549 nm, emission at 565 nm). Thus, we are able to monitor individual vesicle docking events by counting the immobilized fluorescent spots in the TIRF video (Figure 1B). The fusion of the docked v-vesicles onto the SBL is identified by the characteristic two-dimensional diffusion of the fluorescent lipid dyes (Figure 1C). With this setup, we are able to monitor the docking rate, the docking-to-fusion delay, and the fusion efficiency in real-time (Figures 1D–G).

We first examine the rate of v-vesicles docking onto the t-SBL. We find, under our experimental conditions, that ~20 vesicles dock in our viewing area (~55 × ~110 μm) during a 1 min video recording (Figure 1E). It is well-established that SNARE complex formation requires VAMP2, syntaxin 1A, and SNAP-25, and even a single missing component fails to elicit docking and fusion. Thus, as a control, we examine the v-vesicles docking onto t-SBLs prepared without SNAP-25 or without both SNAP-25 and syntaxin 1A. In either case, the vesicles fail to associate with the SBL. Furthermore, to test if vesicle docking is exclusively SNARE-mediated we pre-incubate t-SBL with soluble VAMP2 without the transmembrane domain (VpS). VpS has been frequently used as a competitive inhibitor for SNARE-mediated vesicle docking. We observe no docking in the presence of VpS as well, indicating that the v-vesicle docking to the SBL is SNARE-mediated. Because Ca^2+^-sensor Syt1 is not included at this time, we expect that Ca^2+^ should not affect either docking or membrane fusion. Indeed, when the v-vesicles premixed with 500 μM Ca^2+^ are injected into the flow chamber we do not observe any apparent changes in docking kinetics when compared with the results without Ca^2+^ (Figure 1E).

Next, we take a closer look into the individual docked v-vesicles to find out if membrane fusion happens. We analyze three independent recordings and count a total of 23 fusion events out of 62 docked v-vesicles (~35% fusion efficiency) and 20 fusion events out of 63 docked v-vesicles (~32% fusion efficiency) in the absence and presence of Ca^2+^, respectively (Figure 1F). Furthermore, approximately half of the fusion events occur within the first 20 ms after docking for both cases (Figure 1F and G). Thus, our results show that SNAREs alone are capable of mediating fast vesicle fusion within 20 ms.

### Syt1 increases the probability of fast membrane fusion in the presence of Ca^2+^

Syt1 harbors tandem Ca^2+^-binding C2 domains that are tethered to the vesicle membrane via a transmembrane helix (Perin et al., 1991; Chapman, 2008). Previously, in their seminal study, Sudhof and coworkers used the gain of function and the loss of function mutants of Syt1 to demonstrate that the main function of Syt1 as a Ca^2+^-sensor is to increase the probability of fast membrane fusion in response to the Ca^2+^ signal (Fernández-Chacón et al., 2001).

Having observed that SNAREs alone can mediate fast membrane fusion with the SBL platform, we now ask if Syt1 and Ca^2+^ together enhance the membrane fusion probability as Sudhof and coworkers observed in the neuron. If so, how much enhancement does it have?

**Figure 2:**
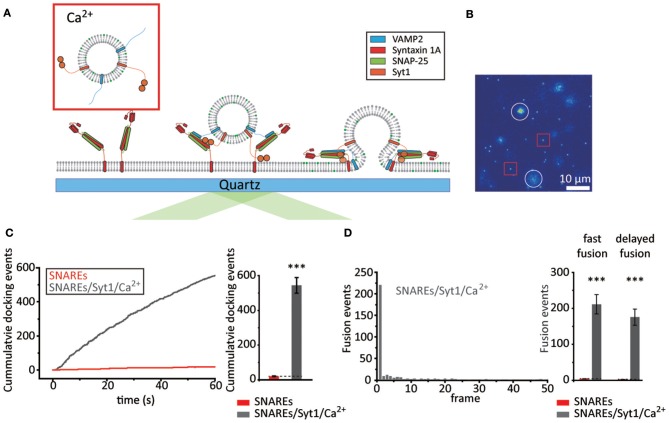
Syt1 increases the probability of fast membrane fusion in the presence of Ca^2+^. **(A)** Schematics for the single vesicle-to-SBL fusion assay with Syt1 and Ca^2+^. **(B)** Screen shot (55 × 55 μm) of the video recording. The red box and white circles indicate representative docked and fusing vesicles, respectively. **(C)** Docking events with v-vesicles reconstituted with both VAMP2 and Syt1 in the presence of 500 μM Ca^2+^ (gray) compared to v-vesicles reconstituted with VAMP2 only (red, also shown in Figure 1E). Representative time trace of cumulative counts of cumulative counts of docking events against time (left) and the number of docked vesicles during the 1 min observation period (right, total of 1,633 docking events from three independent recordings). **(D)** A representative histogram of docking-to-fusion delay distribution from a single 1 min recording (left, each frame is 20 ms). The number of fast and delayed fusion events within a 1 min recording (right). A total of 23 and 1,161 fusion events were observed for SNAREs (red, also shown in Figure 1F) and SNAREs/Syt1/Ca^2+^ (gray), respectively. For **(C)** and **(D)** the data are shown as means ± *SD*. Statistical significance was assessed by Student's *t*-test (^***^p < 0.005; NS, no significant difference; *n* = 3 independent experiments).

To answer these questions, we reconstitute both VAMP2 and Syt1 into the vesicles in a 1:1 ratio. In order to follow through from docking to membrane fusion in a continuous time frame, the vesicles are premixed with Ca^2+^ and the mixture is injected into the flow cell prepared with t-SBL. The vesicle concentration here is set to be equal to that in the previous section, in the absence of Syt1, for the proper comparison (Figure 2A).

We first examine the effect of Syt1 and Ca^2+^ on docking. It is established that Syt1 assists docking either by its t-SNARE interaction (Rickman et al., 2004; Loewen et al., 2006) or by direct binding to negatively charged lipids (Schiavo et al., 1996; Kim et al., 2012). Remarkably, we observe more than ~540 docking events during our 1 min video recordings with 500 μM Ca^2+^, a ~25-fold enhancement over those in the absence of Syt1 (Figures 2B,C, Supplementary Video 1). As for membrane fusion, we find that ~70% of the docked v-vesicles exhibit membrane fusion, which is a 2-fold enhancement when compared with the percentage with SNARE only. Among those, ~55% of the fusion population display docking-to-fusion delay shorter than 20 ms, similar to the case with SNAREs only. In direct comparison with the fusion population observed with v-vesicles without Syt1, we find that Syt1 and 500 μM Ca^2+^ together elicit over a ~50-fold increase in both fast and total fusion population (Figure 2D). Intriguingly, however, most of the enhancement of fast membrane fusion by Syt1 and Ca^2+^ stems from the ~25-fold enhancement of docking while there is only a ~2-fold increase in the fusion-to-docking ratio.

It is often recognized that lipid mixing does not necessarily report the formation of a fusion pore. To make sure that the fusion pore is indeed formed, we prepare v-vesicles that are loaded with both lipid-reporter DiD (excitation at 644 nm, emission at 665 nm) and the content-reporter sulforhodamine B (SRB) (excitation 565 nm, emission at 586 nm) (Kiessling et al., 2013; Stratton et al., 2016) (Supplementary Figure 1A). We are able to monitor both lipid mixing and content release via simultaneous excitation with red (640 nm) and green (532 nm) lasers. We find that v-vesicles showing the lipid diffusion, characteristic to lipid mixing lose the SRB signal instantaneously at the onset of lipid mixing (Supplementary Figure 1B left), while the v-vesicles showing no lipid diffusion maintain their SRB signal (Supplementary Figure 1B right). This implies that lipid mixing and fusion pore opening occur simultaneously within our experimental time resolution of 20 ms. Furthermore, it appears that using DiD instead of DiI or the incorporation of SRB do not significantly affect the overall docking and fusion characteristics (Supplementary Figures 1C,D).

### Dissection of membrane fusion step

**Figure 3:**
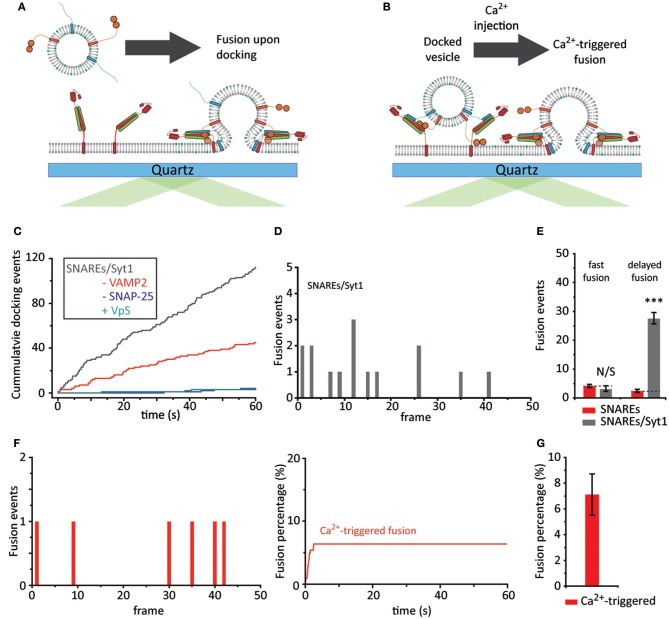
Dissection of membrane fusion steps. **(A)** Schematics for the single vesicle-to-SBL fusion assay with Syt1 and without Ca^2+^. **(B)** Schematics for Ca^2+^-triggered single vesicle-to-SBL fusion assay of a priori docked vesicles. **(C)** Representative cumulative count of docking events against time from one individual recording. The gray trace indicates v-vesicles reconstituted with Syt1 and VAMP2 and the red trace indicates vesicles with just Syt1. Controls without SNAP-25 and with VpS are also shown in blue and green, respectively. **(D)** A representative histogram of fusion events plotted against docking-to-fusion frame delay (20 ms) from one individual recording. **(E)** The number of fast and delayed fusion events. The red bars indicate v-vesicles reconstituted with just VAMP2 (23 fusion events, also shown in Figure 1G) and gray bars indicate v-vesicles reconstituted with VAMP2 and Syt1 (90 fusion events). **(F)** Ca^2+^-triggered fusion events plotted against time. A representative histogram of frame delay (20 ms) between Ca^2+^ injection and fusion within the first 100 s (left) and the cumulative plot during the entire 1 min recording is shown (right). **(G)** The fusion probability of Ca^2+^-triggered events within a 1 min recording. A total of 74 fusion events among 1,106 docked vesicles were observed from three independent recordings. For **(E,G)** the data are shown as means ± *SD*. Statistical significance was assessed by Student's *t*-test (^***^p < 0.005; NS, no significant difference; *n* = 3 independent experiments).

In the experiments presented in the previous section, we premixed Ca^2+^ with the v-vesicles. This experimental preparation mimics the pre-binding of Ca^2+^ to Syt1 prior to Syt1's binding to SNAREs and the target membrane. However, in reality, it is possible that Syt1 pre-binds to SNAREs in the absence of Ca^2+^ in preparation for the Ca^2+^ influx. In fact, it is shown that Syt1 has the capacity to bind the binary t-SNARE complex in the absence of Ca^2+^ (Rickman et al., 2004; Loewen et al., 2006). To explore the outcomes of this alternative situation, we examine docking and fusion in the absence of Ca^2+^ (Figure 3A) and investigate whether Ca^2+^ can trigger membrane fusion of a priorily docked vesicles (Figure 3B).

When we examine vesicle docking in the absence of Ca^2+^ we observe a ~6-fold increase in docking compared to the results from the vesicles with only VAMP2 (Figure 3C). This enhancement factor falls significantly short of those observed when both Ca^2+^ and Syt1 are present, indicating that Ca^2+^ plays a role in vesicle docking.

To our surprise, no apparent accumulation of fast membrane fusion is observed within the first 20 ms time period. This is in sharp contrast to that of which was observed for the experiments with only SNAREs. Membrane fusion events among docked vesicles are all scattered randomly over the period of our experimental time (1 min) (Figures 3D,E). Nevertheless, the results appear to be somewhat consistent with the proposal that Syt1 might function as a fusion clamp of spontaneous fusion (Chicka et al., 2008).

Even more surprising is that the strong enhancement of membrane fusion by Ca^2+^, observed when Ca^2+^ was pre-bound to Syt1, is completely lost. When we inject Ca^2+^ (Figure 3B), after any unbound vesicles are washed out with sufficient amount of buffer, we do not detect any synchronization of membrane fusion events in the first 20 ms window. Mere ~7% of docked vesicles fuses with SBL with 500 μM Ca^2+^ and fusion events are sporadically scattered in the time frame of our observation (1 min) (Figures 3F,G). Thus, the results show that a priori binding of Syt1 to SNARE complexes in the absence of Ca^2+^ leads to an irreversible off-pathway that does not bring about Ca^2+^-triggered synchronized membrane fusion.

**Figure 4:**
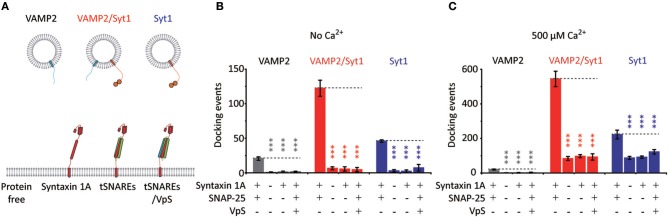
Vesicle docking requires both t-SNAREs, syntaxin 1A and SNAP-25. **(A)** Schematics for the single vesicle-to-SBL docking assay. We prepared v-vesicles reconstituted with VAMP2, both VAMP2 and Syt1, and only Syt1. We separately evaluated docking of v-vesicles onto SBLs reconstituted without proteins, with only syntaxin 1A, with t-SNAREs, and with t-SNAREs disabled by VpS. **(B)** The number of docking events for v-vesicles reconstituted with VAMP2 only (gray), with VAMP2 and Syt1 (red), and with Syt1 only (blue) in the absence of Ca^2+^. **(C)** The number of docking events for v-vesicles reconstituted with VAMP2 only (gray), with VAMP2 and Syt1 (red), and with Syt1 only (blue) in the presence of 500 μM Ca^2+^. For **(B)** and **(C)** the data are shown as means ± *SD*. Statistical significance was assessed by Student's *t*-test (^***^p < 0.005; NS, no significant difference; *n* = 3 independent experiments).

As controls, we evaluate docking and fusion of vesicles reconstituted with both VAMP2 and Syt1, with only VAMP2, with only Syt1, onto SBLs with t-SNAREs, with only syntaxin 1A, with t-SNAREs disabled by VpS, and without any proteins (Figure 4A). Experiments with all possible combinations are separately carried out either in the absence or presence of 500 μM Ca^2+^ in Figures 4B,C, respectively. Even in the absence of VAMP2, we observe docking with v-vesicles reconstituted with only Syt1 (Figure 4B). This is due to Syt1 binding to either the binary t-SNARE complex, the ternary SNARE complex or phosphatidylinositol-4,5-bisphosphate (PIP2) (Rickman et al., 2004; Loewen et al., 2006). Furthermore, it is established that Ca^2+^ increases binding between Syt1 and PIP2 which is shown in Figure 4C (Schiavo et al., 1996; Kim et al., 2012). However, we note that these docking events did not lead to membrane fusion.

## Discussion

SNAREs alone may have the capability to drive fast membrane fusion. SNARE-driven fast fusion in the millisecond time scale was observed in previous studies but the results had caveats: the requirement of SNAP-25 was not met (Liu et al., 2005) and fast membrane fusion was only observed with the addition of a small soluble fragment of VAMP2 (Domanska et al., 2009). More recently, significant fusion enhancement has been observed with Ca^2+^ even in the absence of Syt1, adding further confusion to the issue (Kiessling et al., 2013). However, our experiments display fast membrane fusion that meets the requirement of SNAP-25 and we observe strong competitive inhibition by VpS, confirming that fast membrane fusion is strictly SNARE-dependent. Thus, our results confirm that SNAREs are indeed capable of driving fast millisecond-time scale membrane fusion.

When both Syt1 and Ca^2+^ are incorporated into our system, membrane fusion is dramatically enhanced by as much as ~50-fold. Previously, using gain of function and loss of function mutants, Sudhof and coworkers reported the increase of the release probability by Syt1 with Ca^2+^ without a significant change in the time scale of the release (Fernández-Chacón et al., 2001). Our results are largely consistent with this *in vivo* observation and show that, while SNAREs are responsible for the fusion kinetics, Syt1 and Ca^2+^ plays a role in dramatically increasing the fusion probability. However, caution is warranted due to our instrumental limitation (20 ms acquisition time). Although we have observed accumulation of fusion events within the first 20 ms with Ca^2+^, it is still possible that these Ca^2+^-triggered fusion events happen faster than 20 ms. Further investigations with a faster instrumentation would resolve this issue.

It is however quite intriguing that the enhancement of the membrane fusion events by Syt1 and Ca^2+^ are mainly due to the stimulation of vesicle docking although we observed a factor of two increase of fusion probability for the docked vesicles. Here, one must be careful in interpreting the *in vitro* data because vesicle docking in *in vitro* setup closely correlates with an initial step in SNARE complex formation. It is believed that initial preassembly of the N-terminal half of the SNARE motifs occurs concurrently with vesicle docking. Given that, we speculate that Ca^2+^-activated Syt1 might play a role in promoting the SNARE preassembly at the N-terminal region (Zhou et al., 2015)

Surprisingly, when v-vesicles are allowed to bind to the t-SBL, a priorily in the absence of Ca^2+^, the results are quite different from the aforementioned results. First, the fast membrane fusion that we observed with only SNAREs, without Syt1, disappears. The results show that Syt1 might act as an inhibitor of fast SNARE-mediated membrane fusion. Such “clamping” effects were previously observed by Chapman and coworkers (Chicka et al., 2008). Moreover, the system did not respond to the subsequent addition of Ca^2+^ at all. Thus, it appears that Ca^2+^ binding to Syt1 prior to Syt1's interaction with either t-SNAREs or PIP2, residing on the plasma membrane, may be an essential requirement for the productive Ca^2+^-triggered fast membrane fusion.

**Figure 5:**
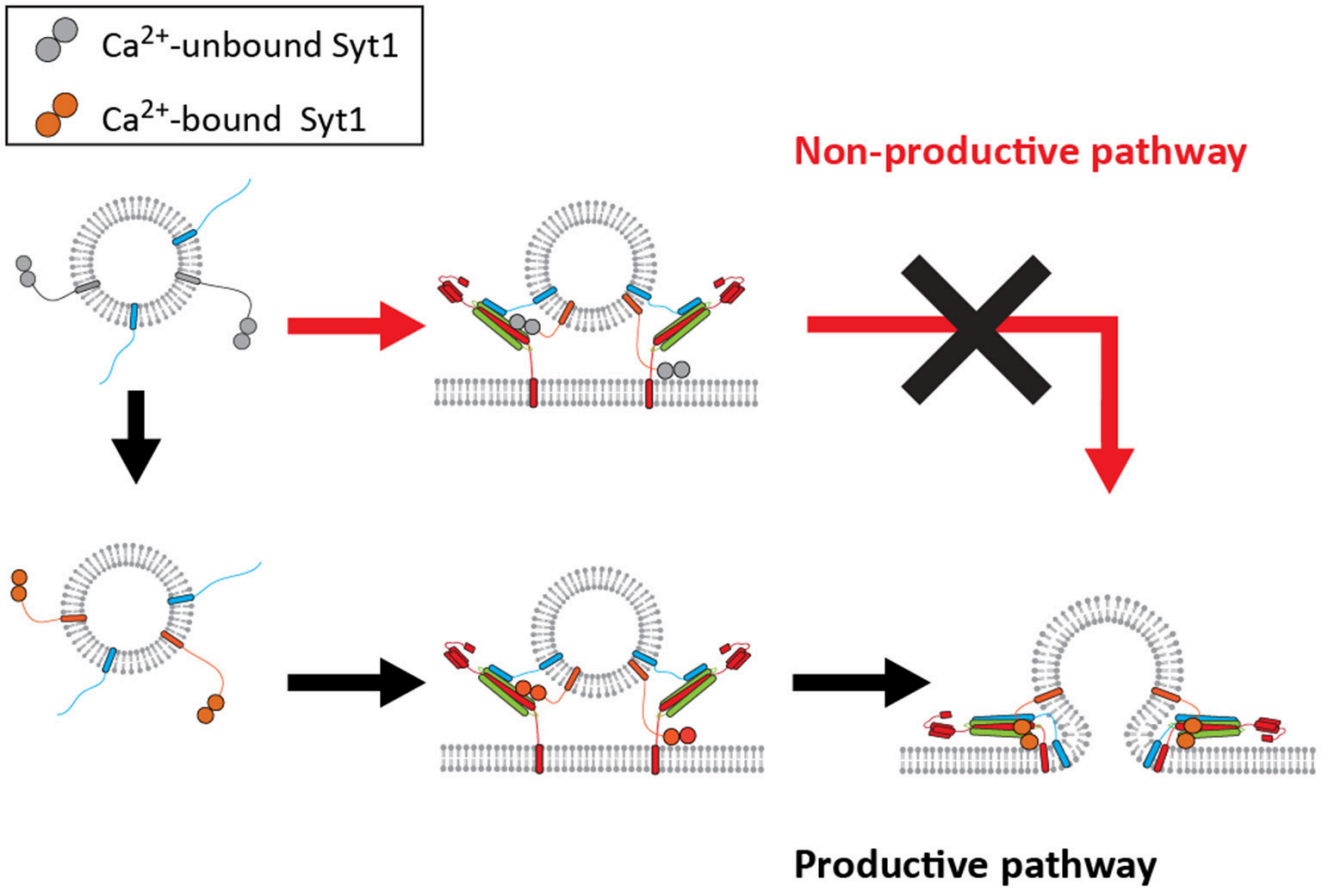
A mechanistic model for productive and non-productive pathway for fast membrane fusion. The productive pathway (black arrows) requires binding of Syt1 with Ca^2+^ prior to its interaction with either t-SNAREs or the lipid membrane for fast membrane fusion. However, the vesicles do not respond to Ca^2+^ if they have already docked, thus being trapped in the non-productive pathway (red arrows).

Taking these observations into consideration we propose two pathways for Syt1: the productive pathway and non-productive pathway. In the productive pathway, shown in black arrows in Figure 5, Syt1 is able to bind Ca^2+^ prior to ternary SNARE complex formation and vesicle docking. This significantly increases the probability of proper SNARE assembly and membrane fusion. However, in the non-productive pathway, shown in red arrows in Figure 5, SNAREs and Syt1 form a premature complex in the absence of Ca^2+^, which results in the failure to respond productively to the Ca^2+^ influx.

How would the proposed non-productive pathway be avoided *in vivo* by Syt1? It is most likely that Syt1 interacts with the binary t-SNARE complex of syntaxin 1A and SNAP-25. In fact, the Ca^2+^-independent interaction between these two has been documented many times (Rickman et al., 2004; Loewen et al., 2006). One strategy to avoid such an inadvertent interaction would be to keep syntaxin 1A and SNAP-25 separated until Ca^2+^ influx. Rizo and coworkers proposed a fusion model where Munc13 and Munc18 choreograph the assembly of the t-binary complex and subsequent SNARE complex formation in a Ca^2+^ dependent manner (Ma et al., 2013; Liu et al., 2016). Alternatively, the Syt1 interaction with the binary t-SNARE complex could be sterically avoided. It is also possible that Munc13 bridges the synaptic vesicle and plasma membrane and enable the formation of a primed state (Liu et al., 2016). If this gap is large enough the non-productive pathway could be avoided. Very recently, Brunger and coworkers proposed that a tripartite SNARE-Cpx-Syt1 complex locks membrane fusion until the arrival of Ca^2+^ (Zhou et al., 2017). However, it remains unclear if the tripartite complex represents the pre- or the post-fusion complex, assuming that the SNARE complex is not fully zippered in the pre-fusion state (Lou and Shin, 2016).

One might ask why fast time scales were not observed in the vesicle-to-vesicle fusion assay. This assay has been widely used to gain insights into SNARE-mediated membrane fusion and functions of the accessory proteins (Lee et al., 2010; Kyoung et al., 2011; Ma et al., 2013; Lai et al., 2014). There may be a bilayer curvature issue in this configuration. Specifically, t-vesicles do not represent the planar presynaptic plasma membrane well. It is possible that fusion between two curved lipid membranes is energetically unfavorable in comparison to fusion between a curved vesicle and the planar membrane, warranting further investigation. More importantly, previous attempts focused on triggering fusion of pre-docked vesicle pairs with the Ca^2+^ injection. This was due in part to the assumption that fusion machinery be pre-assembled prior to the Ca^2+^ influx. However, our experiments show that such pre-assembly of the fusion machinery may not be required and the fast millisecond-time scale membrane fusion can happen even though there are no pre-assembly of the fusion machinery and no Cpx.

## Materials and methods

### Plasmid construct and site-directed mutagenesis

We prepared DNA sequences encoding rat syntaxin 1A (amino acids 1–288 with three native cysteines replaced by alanines), VAMP2 (amino acids 1–116 with C103 replaced by alanines), VpS (amino acids 1-94), SNAP-25 (amino acids 1–206 with four native cysteines replaced by alanines) and inserted them into pGEX-KG vector as N-terminal GST fusion proteins. We also prepared rat Syt1 (amino acids 50-421 with four native cysteines C74, C75, C77 and C79 replaced by alanines and another C82 replaced by serine) and inserted it into pET-28b vector as C-terminal His-tagged proteins. DNA sequences were confirmed by the Iowa State University DNA Sequencing Facility.

### Protein expression and purification

*Escherichia coli* BL21 Rosetta (DE3) pLysS (Novagen) was used to express the recombinant GST fusion proteins VAMP2, SNAP-25, syntaxin 1A and VpS. The cells were grown in LB medium at 37°C with ampicillin (100 μg/mL) until the ~0.6–0.8 absorbance at 600 nm. The cells were further grown for another 12 h at 16°C after induction with the addition of Isopropyl β-D-1-thiogalactopyranoside (0.3 mM final concentration). The cell pellets were then harvested via centrifugation at 6,000 × g for 10 min and resuspended in PBS at pH 7.4 containing 2 mM 4-(2-aminoethyl)-benzenesulfonyl fluoride (AEBSF), 2 mM EDTA, and 2 mM dithiothreitol. For the transmembrane proteins, in addition to the resuspension buffer we added 0.5% Triton X-100. The cells were lysed with sonication immersed in an ice bath. The lysed cells were centrifuged at 15,000 × g for 20 min and the supernatant was added onto columns with glutathione-agarose beads for 4°C for 2 h. The unbound proteins were thoroughly washed off and thrombin (Sigma-Aldrich) was added to cleave off the GST fusion proteins at 4°C for 16 h. We note that the thrombin cleavage buffer was 50 mM Tris-HCl, 150 mM NaCl, and pH 8.0 and additional 1% n-octyl glucoside was added for transmembrane proteins.

The C-terminal His-tagged Syt1 (amino acids 51–421) was also expressed in *E. coli* BL21 Rosetta (DE3) pLysS and was purified identically to the aforementioned protocol with the exception of using Ni-NTA beads (Qiagen) instead of GST beads. Also, the elution buffer was prepared with 25 mM HEPES, 400 mM KCl, 500 mM imidazole, and 0.8% OG. All purified proteins were examined with 15% SDS-PAGE and the purity was at least 90%.

### V-vesicle reconstitution

We used the following lipid molecules to make v-vesicles: 1,2-dioleoyl-sn-glycero-3-phospho-L-serine (DOPS), 1-palmitoyl-2-oleoyl-snglycero-3-phosphocholine (POPC), 1,1′-dioctadecyl-3,3,3′,3′-tetramethylindocarbocyanine perchlorate (DiI, Invitrogen), 1,1′-dioctadecyl-3,3,3′,3′-tetramethylindodicarbocyanine perchlorate (DiD, Invitrogen), and cholesterol. All lipids in this study were obtained from Avanti Polar Lipids if not otherwise specified.

For v-vesicles with just DiI, we first mixed lipids with molar ratios of 5:54:40:1 (DOPS: POPC: Cholesterol: DiI) and dried it into lipid film using gentle nitrogen gas in a glass tube. The lipid film was stored overnight in a desiccator under house vacuum. The lipid film was resuspended with buffer containing 25 mM HEPES 100 mM KCL pH 7.4. We made liposomes with 10 flash freeze-thaw cycles and large unilamellar vesicles (~100 nm in diameter) were prepared by extrusion through the polycarbonate filter (Avanti Polar lipids). VAMP2 and Syt1 was mixed with the vesicles such that the lipid to protein ratio was 200:1. The liposome/protein mixture was diluted by adding three times the volume of the protein lipid mixture and then dialyzed in 2 L dialysis buffer at 4°C overnight.

We also prepared v-vesicles with both lipid-reporter DiD and the content-reporter sulforhodamine B (SRB). These vesicles were prepared identical to the aforementioned v-vesicles with the exception of substituting DiD for DiI. Also, the SRB concentration was kept constant (20 mM) after resuspension of the dried lipid film. Free SRB was removed with the PD-10 desalting column (GE healthcare) after overnight dialysis.

### T-SBL preparation

The lipid film for the t-SBL was prepared identically to the case of v-vesicles with the exception of lipid composition. We used the following lipids: DOPS, POPC, phosphatidylinositol-4,5-bisphosphate (PIP2, from porcine brain), and 1,2-dipalmitoyl-sn-glycero-3-phosphoethanolamine-N-[methoxy(polyethylene glycol)-2000] (PEG2000). We mixed the lipids using the following molar ratio of 15:78:2:5 (DOPS: POPC: PIP2: PEG2000). The lipid film was dried and stored in a desiccator overnight. The lipid film was resuspended with buffer containing 25 mM HEPES 100 mM KCL 1% OG pH 7.4. We mixed the resuspended lipid with t-binary complex which was prepared by mixing syntaxin 1A and SNAP-25 (1:1.5 molar ratio) for 30 min at room temperature. The lipid to t-binary complex ratio was 2000:1. Then the liposome/protein mixture was diluted by adding three times the volume of the protein lipid mixture using 25 mM HEPES 100 mM KCL pH 7.4 and then dialyzed overnight at 4°C in a 2 L beaker.

We subjected the imaging quartz slide to piranha cleaning, boiling mixture of sulfuric and hydrogen peroxide, for 20 min. The slides were rinsed with de-ionized water and placed in a sonicator for 20 min to rid of any residual acid. We quickly assembled the slides and gently injected the t-proteoliposomes into the flow chamber. We let the t-SBL form for 2 h at room temperature, washed out excess liposomes and let the samples settle for 2 h.

### Single vesicle-to-SBL fusion assay

Once the t-SBL was prepared, we mounted the imaging quartz slide on the total internal reflection (TIR) fluorescence microscope. The TIR angle of the laser (532 nm) was properly adjusted and then we initiated the real-time movie acquisition with an imaging area of ~55 × ~110 μm and 20 ms time resolution for 90 s. We then gently injected the v-vesicles (prepared according to the experiment) into the flow chamber until the buffer within the flow chamber was exchanged with the v-vesicle solution. The injection pump was promptly stopped in order to prevent any unwanted fusion events due to the flow effect. Once the 90 s movie was obtained, we analyzed a 60 s segment of the movie starting after the injection pump was stopped with our custom built program.

With our custom-built analysis program we monitored the fluorescent DiI signals (DiD and SRB signals for the experiments in Figures 2E,F) from the v-vesicles in order to determine docking and fusion. First, we scrolled through the entire 60 s movie clip and handpicked every immobilized spot which represents docked v-vesicles. By selecting the immobilized spot with our program we were able to record the x- and y-coordinates as well as the time at which the vesicle docked on to the t-SBL. Using the x- and y- coordinates, the selected spots were visually marked in order to avoid any recounting or omission.

We then re-examined the movie clip and looked for the characteristic two dimensional diffusion of the fluorescent lipids. The docking-to-fusion delay time was determined by counting the number of frames between immobilization and two dimensional expansion of the fluorescence signal. Thus, the fast fusion events (sub 20 ms fusion delay time) displayed expansion of the fluorescence signal in the absence of a distinct immobilization step. From the analysis process we were able to monitor individual v-vesicles and obtained the docking rate, the fusion percentage, and the fusion delay time between docking and fusion within a movie clip. This analysis process was executed on each movie recording. Three independent recordings were analyzed to obtain the statistics and the statistical significance for each data set.

## Author contributions

JK and YKS designed the experiments. JK performed the experiments. JK and YKS wrote the paper.

### Conflict of interest statement

The authors declare that the research was conducted in the absence of any commercial or financial relationships that could be construed as a potential conflict of interest.

## Supplementary material

Supplementary Figure 1 Accompanying Figure 2, single vesicle-to-SBL fusion assay with v-vesicles loaded with both fluorescent lipid dye DiD and content dye SRB. **(A)** v-vesicles loaded with the fluorescent lipid dye DiD as well as the content dye SRB for simultaneous monitoring of lipid mixing and content release. **(B)** Representative time traces of DiD lipid dye (red) and SRB content dye (green) of membrane fusion (left) and just docking (right) are shown. **(C)** Number of docking events with v-vesicles reconstituted with VAMP2/Syt1 in the presence of 500 μM Ca^2+^ (left). Due to the rise in background signal from the released content dye, we analyzed the first 30 s of the 1 min recording and reduced the v-vesicle concentration 2-folds compared to the experiments performed in Figure 2. The docking events for v-vesicles reconstituted with just VAMP2 are shown in the middle. We increased the vesicle concentration 20-folds (indicated by the red arrow) in order to observe a comparable number of docking events. A bar graph of the normalized docking events taking the vesicle concentration into consideration is also shown (right). **(D)** The percentage of fast and delayed fusion events observed among total docked vesicles (left). A bar graph of the normalized fusion events taking the vesicle concentration into consideration is also shown (right). A total of 66 and 249 fusion events were observed for SNAREs (red) and SNAREs/Syt1/Ca^2+^ (gray), respectively. For **(C)** and **(D)** the data are shown as means ± *SD*. Statistical significance was assessed by Student's *t*-test (^***^*p* < 0.005; NS, no significant difference; *n* = 3 independent experiments).

Supplementary Video 1 Accompanying Figure 2, a representative movie acquisition of docking and fusion of v-vesicles with VAMP2, Syt1, and Ca^2+^ on t-SLB. This movie is a 10 s excerpt from a 1 min recording filmed at 50 frames per second (fps) and played at 50 fps.
