# Supplementary figures and images for: Productive and Non-productive Pathways for Synaptotagmin 1 to Support Ca2+-Triggered Fast Exocytosis
Source: Front Mol Neurosci. 2017 Nov 15;10:380. doi: 10.3389/fnmol.2017.00380 (PMC5695160; doi:10.3389/fnmol.2017.00380)

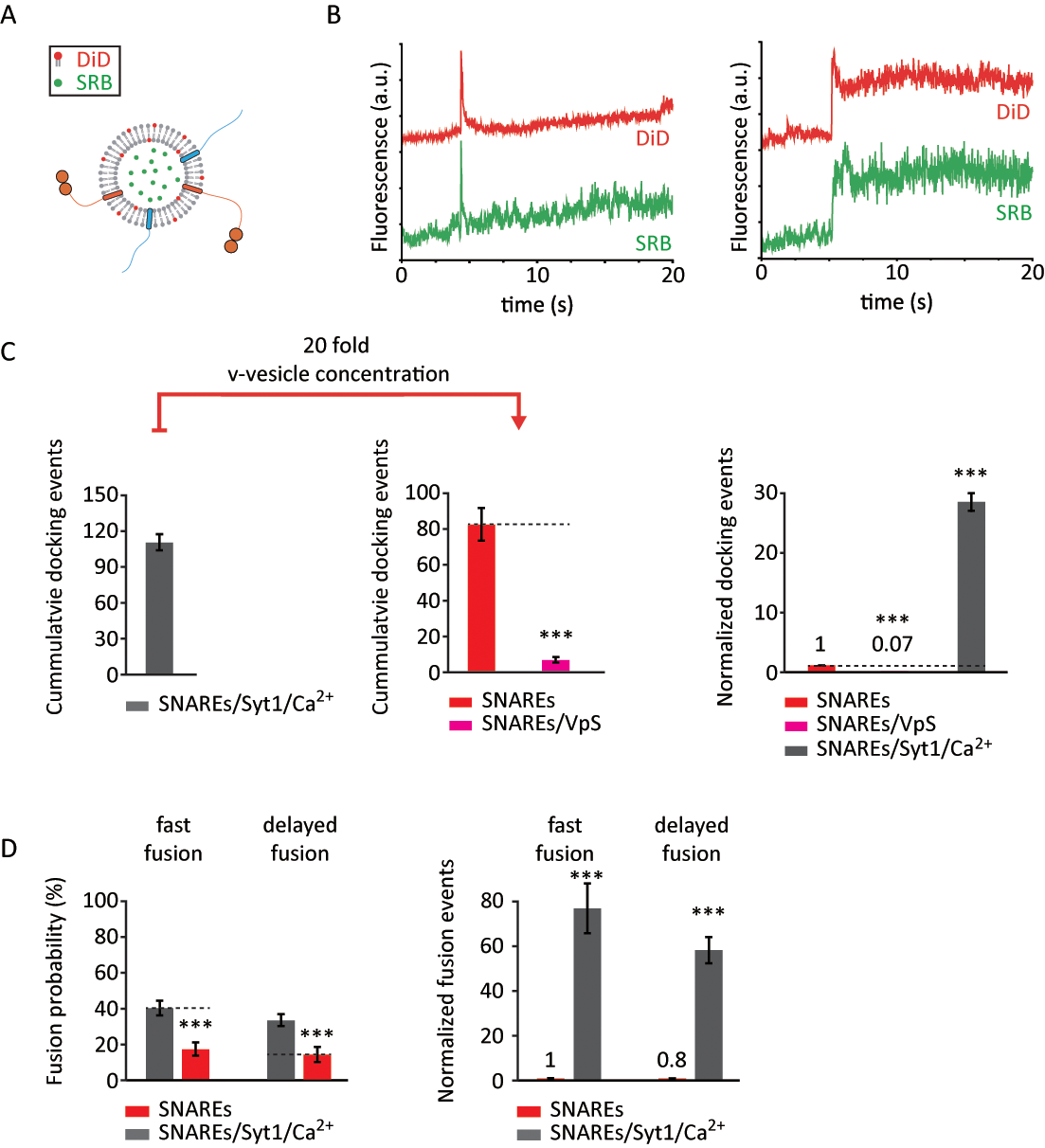

Supplement: Supplementary Figure 1 — Accompanying Figure 2, single vesicle-to-SBL fusion assay with v-vesicles loaded with both fluorescent lipid dye DiD and content dye SRB. (A) v-vesicles loaded with the fluorescent lipid dye DiD as well as the content dye SRB for simultaneous monitoring of lipid mixing and content release. (B) Representative time traces of DiD lipid dye (red) and SRB content dye (green) of membrane fusion (left) and just docking (right) are shown. (C) Number of docking events with v-vesicles reconstituted with VAMP2/Syt1 in the presence of 500 μM Ca2+ (left). Due to the rise in background signal from the released content dye, we analyzed the first 30 s of the 1 min recording and reduced the v-vesicle concentration 2-folds compared to the experiments performed in Figure 2. The docking events for v-vesicles reconstituted with just VAMP2 are shown in the middle. We increased the vesicle concentration 20-folds (indicated by the red arrow) in order to observe a comparable number of docking events. A bar graph of the normalized docking events taking the vesicle concentration into consideration is also shown (right). (D) The percentage of fast and delayed fusion events observed among total docked vesicles (left). A bar graph of the normalized fusion events taking the vesicle concentration into consideration is also shown (right). A total of 66 and 249 fusion events were observed for SNAREs (red) and SNAREs/Syt1/Ca2+ (gray), respectively. For (C) and (D) the data are shown as means ± SD. Statistical significance was assessed by Student's t-test (***p < 0.005; NS, no significant difference; n = 3 independent experiments). [file Image1.TIF]
